# Mitochondrial Cristae as Separate Compartments: Linking Organization and Function

**DOI:** 10.3390/cimb48080792

**Published:** 2026-08-04

**Authors:** Alexander V. Panov, Semen V. Nesterov, Lev S. Yaguzhinsky

**Affiliations:** 1 Department of Biomedical Sciences, School of Medicine, Mercer University, Macon, GA 31201, USA; 2 Moscow Center for Advanced Studies, Kulakova Str. 20, 123592 Moscow, Russia; 3 Belozersky Research Institute for Physico-Chemical Biology, Lomonosov Moscow State University, 119992 Moscow, Russia

**Keywords:** mitochondrial cristae, microcompartmentalization, nonequilibrium thermodynamics, proton motive force, transhydrogenase, NADPH transport, NADPH-isocitrate dehydrogenases, cellular bioenergetics

## Abstract

Traditional bioenergetic paradigms historically relied on classical equilibrium thermodynamics to calculate mitochondrial kinetics, often overlooking the non-equilibrium processes dictated by complex structural architecture. Recent discoveries fundamentally challenge these outdated views by demonstrating that the inner mitochondrial membrane is strictly segregated into distinct functional domains, where individual cristae operate as autonomous, ultra-confined nanocompartments, where the transport of metabolites and protons is tightly controlled by ultrastructure-assisted electric and entropic effects. Compartmentalization prevents proton dissipation, allows for the rapid generation of a localized proton motive force optimized for efficient ATP synthesis and provides robust functional redundancy against localized membrane damage. Furthermore, recognizing cristae as isolated microspaces resolves the long-standing paradox of mitochondrial nicotinamide adenine dinucleotide transhydrogenase (TH). We describe a multi-stage transport pipeline—the TH–isocitrate dehydrogenase axis—wherein matrix-generated reducing equivalents are exported into the cytoplasm via an irreversible isocitrate/α-ketoglutarate loop. This universal pipeline continuously supplies uncommitted NADPH for biosynthesis, systemic antioxidant defense and detoxification. We also highlight the role of compartmentalization in ATP transport and utilization processes. Consequently, disruptions to cristae compartmentalization emerge as primary pathogenic drivers in ischemic, neurodegenerative, and cardiovascular diseases.

## 1. Mitochondrial Compartmentalization

Since the early days of mitochondriology, it has been thought that the folding of the inner mitochondrial membrane (IMM) was necessary simply to accommodate as many oxidative phosphorylation (OXPHOS) enzymes and other functional proteins as possible. These IMM folds, called “cristae,” were historically thought to result from the passive packing of a large IMM surface area into the small, confined space defined by the outer mitochondrial membrane (OMM). However, discoveries in recent decades have fundamentally changed our understanding of cristae formation, their functions, and mitochondria in general.

The surface area of the IMM is several times larger than that of the OMM, which strictly limits the internal volume of the mitochondrion. This volume can change dynamically as a result of the fusion and fission of individual mitochondria, processes that depend directly on the energy state of the organs. In humans, the smallest mitochondria are found in the cerebellum and cerebral cortex, whereas the largest mitochondria reside in the renal cortex, heart, and skeletal muscles. The size of the host animal also affects both the size and total number of mitochondria [1].

**Figure 1:**
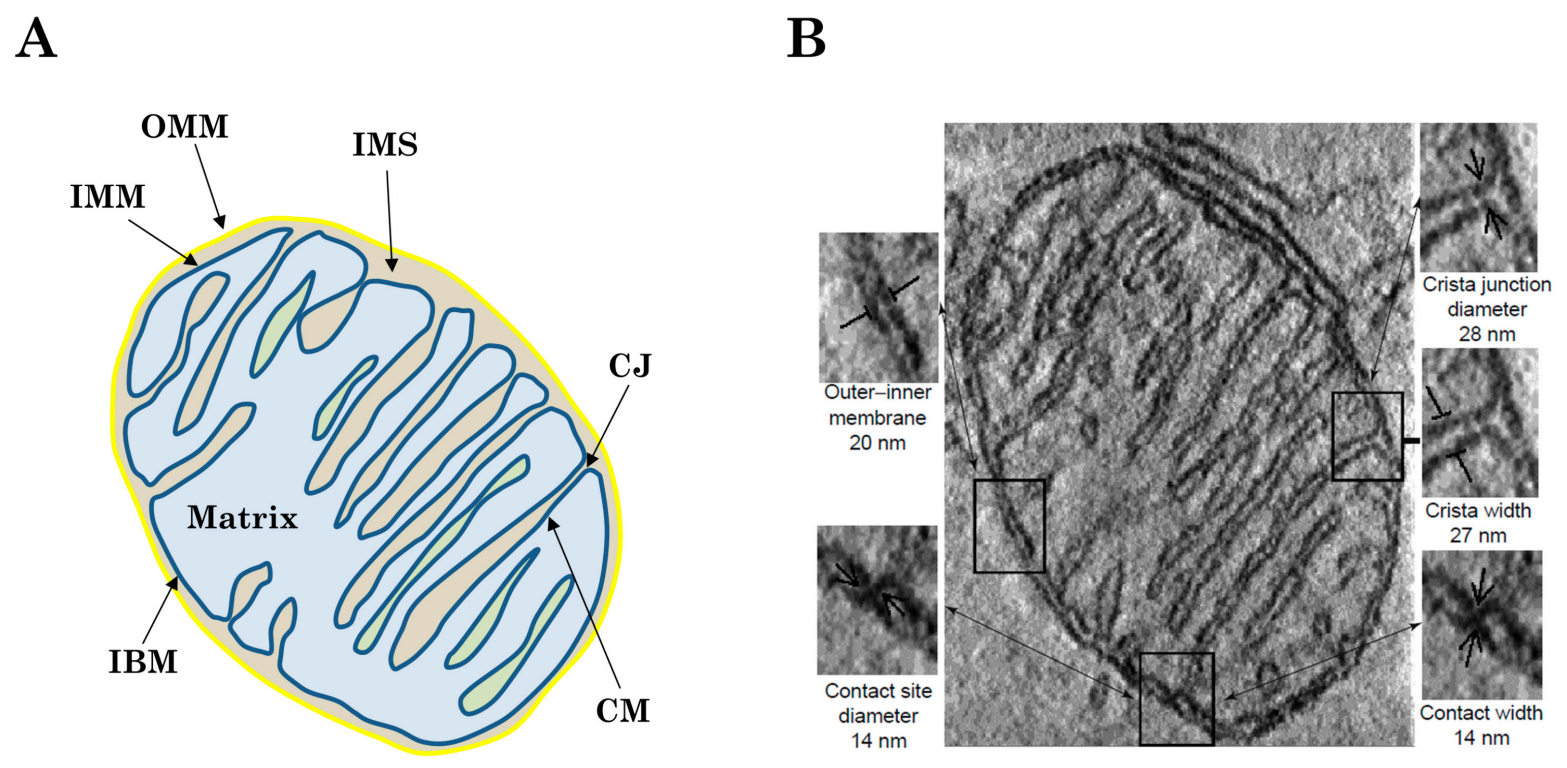
Old ideas about the structure of mitochondrial microcompartments. (**A**) Schematic representation of mitochondrial architecture. The outer mitochondrial membrane (OMM), inner mitochondrial membrane (IMM), inner boundary membrane (IBM), cristae junctions (CJ), intermembrane space (IMS), cristae membrane (CM) and mitochondrial matrix are indicated. (**B**) Electron micrograph from a cryo-cut mitochondrion with antibody probing of OXPHOS complexes. The localization in the cristae membrane is obvious. Inset: detailed view of the two IMM compartments CM and IBM connected by the cristae junction (CJ). Scale bar: 150 nm. Reproduced from [3].

**Figure 2:**
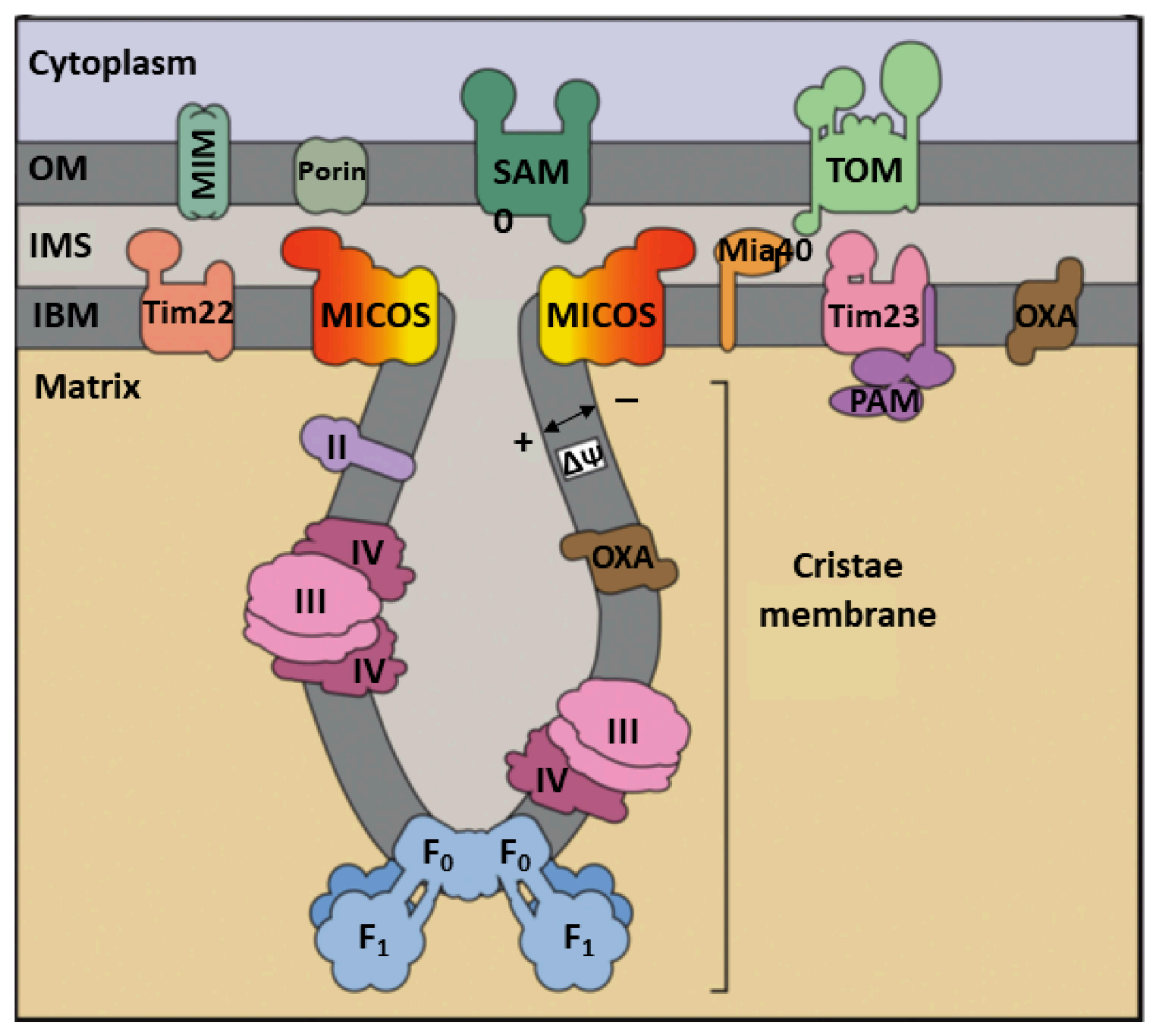
Architecture of the mitochondrial inner membrane. The inner membrane consists of the inner boundary membrane (IBM) and the folded cristae membranes. Crista junctions connect the IBM and cristae. The MICOS complex is enriched at crista junctions. Protein translocases of the inner membrane (TIM22 and TIM23) are preferentially located in the IBM, whereas respiratory chain complexes (I, II, III, IV) and the F1F0-ATP synthase are enriched in cristae membranes. The preferential location of the oxidase assembly (OXA) translocase depends on growth conditions and the availability of substrate proteins. This figure was adapted from [2].

The IMM can be divided into two distinct domains or regions (Figure 1). Part of the IMM runs parallel to and at a short distance from the outer membrane. This region is called the “inner boundary membrane” (IBM) and harbors many protein translocases and transporters, including the calcium uniporter [2]. Other regions of the IMM form invaginations of various lengths and shapes. These regions are called the “cristae membrane” (CM) and carry the respirasomes (supercomplexes of the respiratory chain) and the dimers of ATP synthase [2] (Figure 2).

At the points of contact between the outer and inner membranes, a structural bottleneck named the “cristae junction” (CJ) separates the boundary and cristae membrane domains of the IMM. These cristae junctions are highly complex structures made of numerous proteins that are remarkably conserved, originating from the Gram-negative protobacterium that was the evolutionary precursor to modern mitochondria. This narrowed site of the cristae junction is organized by a group of proteins called the mitochondrial cristae organizing system (MICOS), which also contacts outer-membrane proteins to form the contact sites between the outer and inner membranes [2,4]. Other important parts of CJ structure and regulation are the OPA1 protein [5] and the MICU1 (Mitochondrial Calcium Uptake 1) protein. The last not only coordinates mitochondrial Ca^2+^ flux, but also plays a structural role by stabilizing cristae junctions through its polybasic domain and its interaction with the MICOS complex [6]. Dimers and oligomers of ATP synthase contribute to the formation of the ends and crests of the cristae, generating a negative membrane curvature. The chains of ATP synthase are located on these crests, running in parallel with strings of respirasomes and individual respiratory chain supercomplexes [7].

It is assumed that the cristae junctions serve as barriers to the diffusion of proteins and small substrate molecules from the intermembrane space into the volume of the crista. Therefore, cristae and their internal spaces can be considered as separate microcompartments that provide optimal conditions for the chemiosmotic coupling of O_2_ consumption and ATP formation [8]. The diffusion of ATP is limited in mitochondria [9], at least in part due to the topology of mitochondrial cristae [10].

Cardiolipin constitutes approximately 20% of the total phospholipid content in the IMM [11]. However, it is important to note that about 80% of this total cardiolipin pool is located in the inner leaflet of the IMM. The rest of the cardiolipin is distributed between the outer leaflet of the IMM and the outer membrane. From the above discussion, the following structurally and functionally discernible mitochondrial compartments can be distinguished:

(1) **The outer membrane (OM)** forms the structural boundary of the mitochondrion and contains integral β-barrel proteins. These are structurally and functionally distinct from the integral α-helical proteins of the inner membrane (IM). While the water-filled channels of β-barrel proteins render the OM highly permeable to water and small hydrophilic molecules, the -helical proteins of the IM strictly restrict water passage. Chemically, the OM is characterized by low cardiolipin content, but it is rich in sphingolipids and capable of accumulating cholesterol, a phenomenon that becomes especially pronounced during aging.
(2) **The inner mitochondrial membrane (IMM)** is functionally and structurally segregated into two distinct domains: the inner boundary membrane (IBM), which runs parallel to the OM, and the invaginated cristae membrane. Key differences between these IMM domains include the following: 1. The inner leaflet of the cristae membrane contains the vast majority of mitochondrial cardiolipin, whereas the IBM is relatively depleted of it [12,13]. 2. The IBM and the cristae membrane harbor entirely different sets of functional protein complexes, reflecting their specialized physiological roles [14].
(3) **The boundary intermembrane space (BIMS)** is the peripheral compartment, which is strictly limited by the outer membrane and the inner boundary membrane (IBM). This specific region sequesters cytochrome *c*, protein components involved in apoptosis activation, ions, and various metabolites; it also serves as the structural hub where the transport systems of the OM and IM physically interact. Within this space, densely packed proteins form organized macromolecular networks. In heart mitochondria, for instance, these structures manifest as pseudocrystalline clusters composed of tens or even hundreds of densely packed octamers of mitochondrial creatine kinase (mCK) [15].
(4) **The Intracristal Space (ICS, Cristae Lumen)**: According to traditional, outdated paradigms, the intracristal space was erroneously equated with a generic “intermembrane space” that freely communicates with the cytoplasm via the water-conducting β-barrel channels of the outer membrane to optimize oxidative phosphorylation. In reality, it is into these restricted cristae spaces that the respiratory chain proton pumps translocate protons. The local concentration of protons is exceptionally high in regions enriched with cardiolipin, which directly corresponds to the areas of negative curvature of the cristae membrane. It is precisely within these specialized domains that the cristae membrane organizes respirasomes, fatty acid–oxidation complexes, ATP synthase dimers, and other core enzymes of the OXPHOS system [7,16,17].
(5) **The matrix space** is the internal core bounded by the inner mitochondrial membrane. Due to an exceptionally high protein concentration—particularly evident in the mitochondria of the kidneys, brain, and heart—the matrix exists not as a simple solution, but as a highly dense, viscous gel [18]. Consequently, while this dense gel physically restricts certain rapid metabolic activities, it serves a vital mechanical function by protecting the delicate structure of the cristae from structural damage. Furthermore, the narrow layer of structured H_2_O confined between the IMM and the matrix gel creates a specialized microenvironment that prevents protons and metabolites dissipation [19], thereby maintaining the high local concentrations necessary for efficient enzyme and transmembrane transporter function.
(6) **Liquid Condensates Within the Matrix (Mitochondrial Nucleoids and RNA Granules):** Although traditionally categorized simply as components of the matrix, these entities are actually highly dense, phase-separated, membraneless condensates that can be distinctly visualized via cryo-EM [20]. Nucleoids contain mtDNA associated with mitochondrial transcription factor A (TFAM), whereas RNA granules primarily house the RNA transcripts and the protein synthesis apparatus. Given our rapidly expanding understanding of the roles and operational mechanisms of these phase-separated structures, it is empirically justified to treat them as distinct, autonomous microcompartments within the matrix [21,22].

## 2. Microcompartmentalization as the Mechanism for Promotion of Oxidative Phosphorylation

From the available new data, we have concluded that each crista is an independent space, bounded by a specific region of the inner membrane called the crista membrane, which is distinct from the inner boundary membrane and separated from it by the MICOS protein complexes. It is suggested that the only exits from the cristae space are the contact points between the outer and inner membranes formed by MICOS proteins, VDAC (Voltage-Dependent Anion Channels), and some other proteins, which we will discuss later. Therefore, let us ask ourselves the following question: what are the benefits of a cristae system designed in the form of separate microcompartments? The answer to this question comes from the structure of plant thylakoids.

A single thylakoid is a structure in which photophosphorylation in green plants and cyanobacteria takes place. The first step, photolysis, splits water into hydrogen and oxygen and requires the energy of four photons: 2H_2_O + 4 hν → 4H^+^ + O_2_; the second step, CO_2_ fixation, takes place in the stroma, outside the thylakoids.

**Figure 3:**
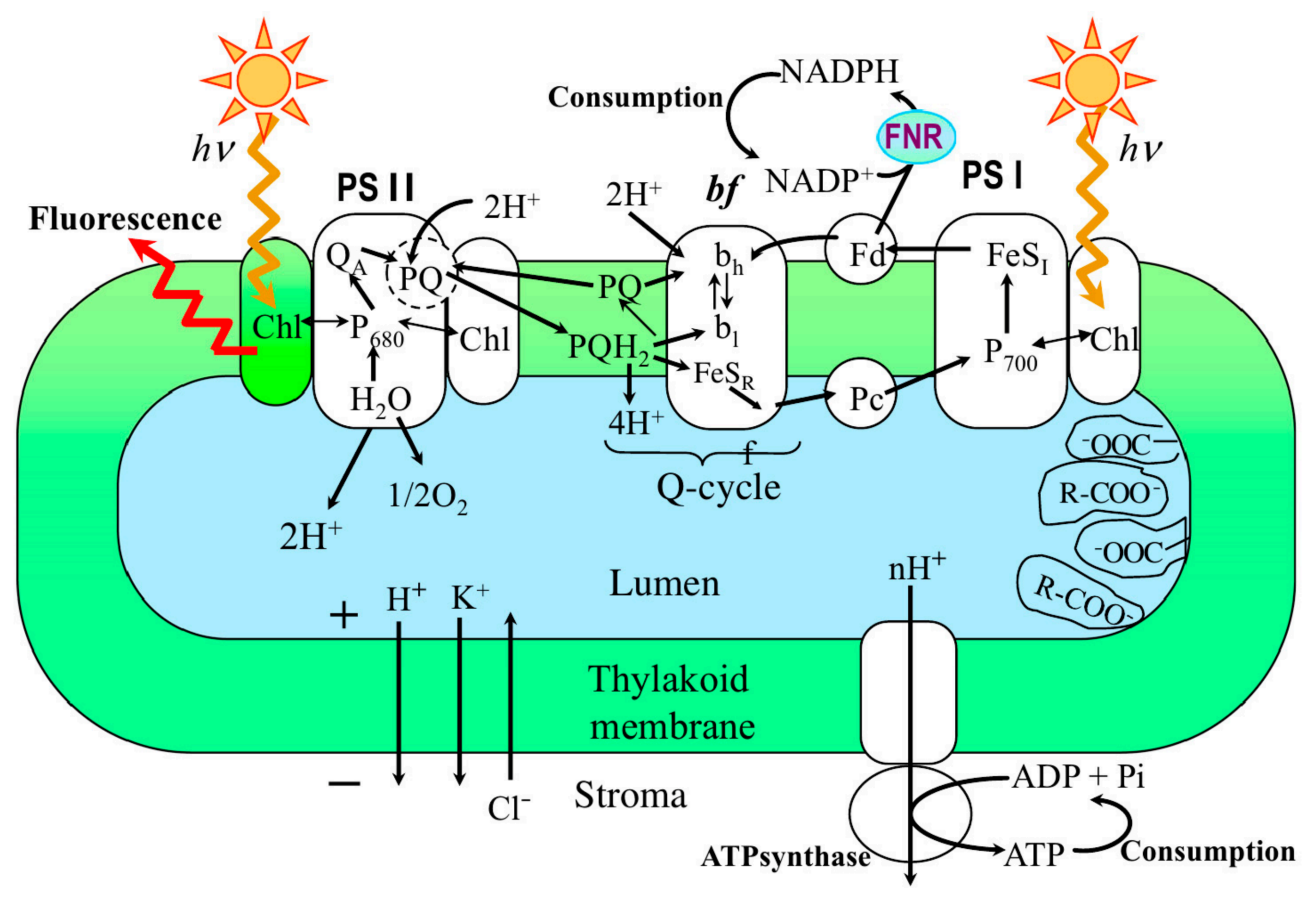
Diagram of the structure of an individual thylakoid. This figure shows the components of the electron transport chain in the chloroplast of a leaf or algae: thylakoid compartments and charge fluxes induced by light. PSII, PSI—photosystems II and I; PQ(PQH2)—plastoquinone (quinol); bf—Cytochrome b-6-f complex; mobile Fd—ferredoxin; Pc—plastocyanin; and R–COO− are buffer groups. Stromal phase components: NADP^+^—NADP^+^, oxidized form; FNR—ferredoxin-NADP-oxidoreductase; and ATP synthase—CF1–CF0 ATP–synthase complex, responsible for the formation of ATP from ADP and inorganic phosphate (Pi). This figure is adapted from [25].

The main idea of Figure 3 that we want to convey to the reader is that photophosphorylation occurs in thylakoids that have a very small volume of the internal space called “lumen”. The entire chloroplast has a volume of ~20–40 μm^3^—10–20% of this volume is occupied by the total volume of thylakoid lumens of ≈2–8 μm^3^, and the volume of the lumen of one thylakoid is ≈0.00073 μm^3^. That is, the total volume of thylakoids in the chloroplast, equal to ≈8 μm^3^, is provided by ≈11,000 thylakoids. When calculating the volume of the internal space (lumen) of the thylakoid, we used the following parameters: thylakoid membrane thickness is 5.1 ± 0.3 nm, thylakoid disk diameter is 300 nm, and total thylakoid thickness is 21.1 ± 1.8 nm. The volume of the thylakoid is as follows: V = S × h, or πR^2^ × h = 0.0007 μm^3^, where R is the radius of the thylakoid (grana) = 145 nm, and h is the height of the inner space (lumen) of the thylakoid (21.1–2 × 5.1) [23,24], π≈3.14.

Thus, efficient photosynthesis in the chloroplast occurs within the extremely small volume of the thylakoid lumen. It is difficult to imagine that photons could generate the necessary potential to split H_2_O into 2H^+^ and O_2_ in a large, unrestricted volume.

The relevant parameters for mitochondria known in the literature are as follows: length: ~1–2 micrometers (μm); diameter: ~0.5–1 micrometer (μm); volume: usually about 0.5–1 μm^3^ for a single mitochondrion. Of course, these data are very approximate, since the size of mitochondria varies greatly depending on the specific organ. So, if we assume that the volume of one mitochondrion is 1 μm^3^, then how can we imagine this volume? Let us compare it with the understandable and well-known volume of 1 microliter, that is, 1 mm^3^. So, in a volume of 1 mm^3^ = 1 μL, theoretically, it would be possible to place 1,000,000,000 μm^3^ = 10^9^ mitochondria!

Typical parameters of the mitochondrial crista are as follows: the width of a crista (lamellar space) is 15–30 nm; crista junction—10–20 nm; membrane thickness—7–10 nm (of each membrane layer); the length of the crista is from several hundred nm, to ~1000 nm; and membrane spacing ~20 nm. Let us try to calculate the approximate volume of the crista by assuming that it is cylindrical in shape, although in reality the crista has a sharp edge at the location of the ATP synthase chain, and assuming the length of the crista as high as 1000 nm.

V = πR^2^ × L = 0.000314 μm^3^, where R is the radius of the crista, which is 10 nm, and L is the length of the crista, which is 1000 nm.

Even assuming minor variations or errors in these calculations, they clearly demonstrate that the water volumes within mitochondrial cristae and thylakoid lumens are of the same structural order and are extraordinarily small. This means that each individual crista can rapidly establish the necessary electrochemical conditions for ATP production. Furthermore, localized damage to a single crista will not significantly impair the overall capacity of the mitochondrion to maintain its membrane potential and perform energy-dependent functions. According to the old paradigm, any damage to the inner membrane causes global catastrophic failure to mitochondrial energetics; the new paradigm of separate microcompartments completely refutes this view.

## 3. Evolution of the Hypothesis of Microcompartmentalization of the Internal Space of the Cristae: From Mechanistic to Kinetic Coupling

In light of the above information, data regarding the key role of IMM phospholipids, particularly cardiolipin, in the functional compartmentalization of cristae are of particular interest, as this compartmentalization appears to be a decisive condition for effective oxidative phosphorylation. The ideas of microcompartmentalization in mitochondria were proposed long ago, based on logical and stoichiometric considerations [26]. The difference in the protein composition of the inner boundary membrane and the crista membrane, as well as the possibility of dynamic remodeling of the crista structure, have also been demonstrated for a long time [27]. An addition to the cristae partitioning hypothesis, suggesting even greater compartmentalization for cardiac mitochondria, was put forward in 2018 and is briefly described below [16].

**Figure 4:**
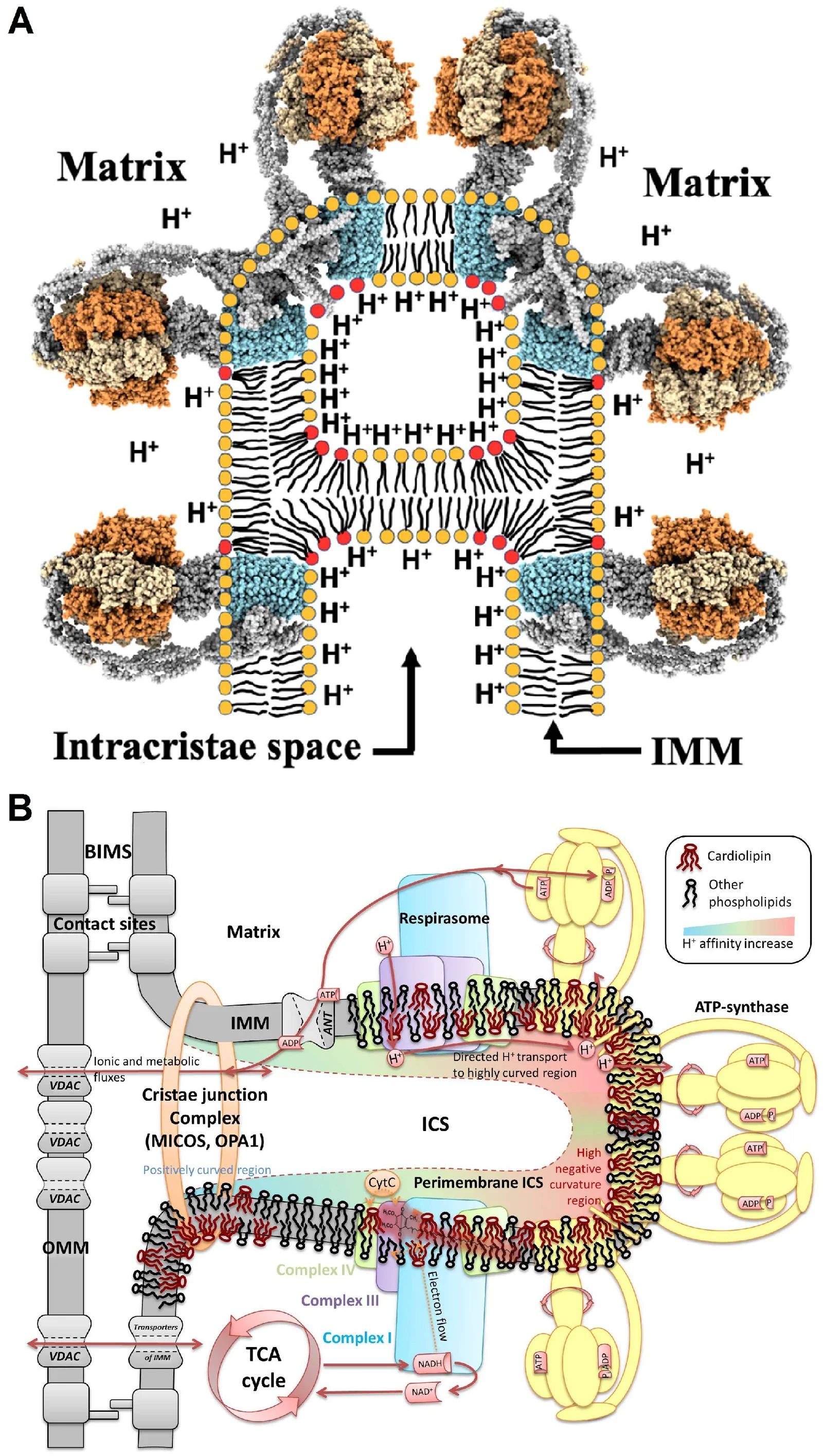
Schematic representation of the proposed cristae compartmentalization models. (**A**). The model based on inverted micelle formed by “non-bilayer” lipids between adjacent leaflets of the IMM of a crista. This figure is derived from [28]. (**B**). The model based on perimembrane protons, showing different affinity for curved membrane regions. The center of ICS still can be used for the diffusion of metabolites in less crowded spaces, while protons directly move to ATP synthase dimers (negative membrane curvature) and do not leave the ICS due to the positive curvature near the cristae junction. “Non-bilayer” lipids are required to stabilize strongly curved areas.

Membrane phospholipids with limited lateral mobility are usually associated with various mitochondrial proteins, forming complexes termed “proteolipids.” These proteolipids are capable of organizing a specialized phospholipid packing architecture within specific regions of the IMM, resulting in the formation of a monomolecular layer of immobilized phospholipids. Mechanistically, this structure behaves like an inverted micelle that physically connects the inner leaflets of the crista membrane (see Figure 4A).

It should be noted that a model utilizing non-bilayer packed lipid bridges between distinct regions of the IMM, as proposed by Gasanov et al., is largely redundant for most types of mitochondria [28]. As calculated above, classical bulb-like cristae possess an internal volume that is already sufficiently small to achieve optimal proton concentration without requiring additional sub-compartmentalization. However, in cardiac mitochondria, the IMM often does not form simple, isolated, bubble-shaped cristae; instead, it develops a highly complex network where lamellae continuously transition into tubular structures and vice versa [29,30]. In such interconnected networks, additional proteolipid separators may, at first glance, seem necessary to maintain localized functional domains.

However, Cryo-ET has clearly demonstrated that in typical cardiac mitochondria, there are no lipid bridges spanning the entire volume of the crista intermembrane space. Instead, reconstructions obtained by cryo-ET reveal a completely different picture: mitochondrial cristae are characterized by extremely high and localized membrane curvature, and this curvature is strictly ordered and associated with the location of large protein complexes. The maximum negative curvature is observed in the vicinity of oligomeric rows of ATP synthase dimers, while the vicinity of respirasomes has a saddle-like shape [30]. This structural feature has enormous functional consequences. It allows us to abandon the need for stable intermembrane bridges for compartmentalization and propose an alternative, more physically sound mechanism. Instead of creating “walls” inside the crista, which inevitably also impede the diffusion of necessary metabolites, nature uses the topology of the membrane surface itself, restricting the movement of hydrogen ions by the laws of electrodynamics (Figure 4B).

As is known, due to the Grotthuss mechanism, the conductivity of water for protons is an order of magnitude higher than for other ions. The internal volume of the crista, filled with water molecules, is an effective conductor for protons. On the surface of a conductor (which is ICS water), according to Gauss’s law (Maxwell’s first equation), charges are extruded from the bulk phase to the surface. Moreover, charges are distributed in such a way that their density is maximal at points with the greatest positive curvature and minimal at points with negative curvature of the conductor surface. It should be emphasized, to avoid confusion, that the ICS bulk phase and IMM have opposite curvature. In Figure 4B, the curvature is shown in relation to the membrane, because this is more visually understandable (the expanding surface area of the membrane layer denotes the positive curvature region). This consequence of the cristae bend has already been noted in the literature [31]. The displacement of protons in non-equilibrium conditions to the interface boundary in model and biological membranes is also a well-proved experimental fact (see [32,33] for review). In more detail, the non-equilibrium proton fraction on the membrane interface is attributed to the entropic effects of water [34,35]. It is very important to note that this property is manifested only when there is an excess of protons in nonequilibrium conditions, when they do not have a counter-ion (in mitochondria during the active operation of proton pumps). This should not be confused with the equilibrium conditions in which the Gouy–Chapman layer is formed.

In relation to mitochondria, this means that protons pumped into the ICS will stay on the membrane surface (in the vicinity of the lipid heads) and will be forced (by the local electric field) to move laterally towards areas of negative membrane curvature, i.e., to the cristae apices where ATP synthase dimers are located, and, conversely, will not move towards the plane areas, or especially to junctions of the crista. By controlling the membrane curvature in this way, even without additional bridges, it is possible to localize protons in the required restricted area. Therefore, the role of such complexes as MICOS or OPA1 lies not in the mechanical blocking of proton diffusion, but in the creation of negative membrane curvature, where the surface charge density will be low. In heart mitochondria, the regions of lamellar–tubular transition will serve the same function as cristae junctions. This rather simple physical principle (curvature-dependent charge distribution on the surface of the conductor) also explains the evolutionary prevalence of ATP-synthase dimerization/oligomerization and conserved saddle-like form of respirasomes [30] mainly provided by the contact of complex I and complex III dimer [36].

It is important to note that this model does not contradict the data obtained by NMR, but rather explains them. The non-bilayer lipid phases observed by Gasanov play an important role in the cristae architecture. Cardiolipin, which is prone to forming non-bilayer structures, concentrates precisely in those places where high curvature is required, and its interaction with ATP synthase dimers stabilizes highly curved membrane regions. Thus, non-bilayer packing of cardiolipin acyl chains is achieved in the local environment of ATP synthases and assists the proton flow. Taking into account the perimembrane localization of protons, the energization of mitochondria dramatically changes the local pH in the perimembrane zone (a potential of 180 mV corresponds to about 3 pH units, while real H^+^ activity may be even higher in innermost region of the electric double layer—the Stern layer), increasing protonation of the phosphate groups of cardiolipin in the vicinity of respirasomes and ATP synthases. This causes cardiolipin to change its packing, creating even greater local curvature of the membrane and thereby forming a more pronounced “channel” for the directed movement of protons [37]. At the distances of several nanometers, H^+^-directed hopping over cardiolipines can be interpreted as the kinetic coupling of the oxidative phosphorylation system [38].

Kinetic coupling—the directed transport of protons along the membrane from pumps to ATP synthases—is essential for mitochondrial function. While calculating proton transfer rates is complex, simple models can estimate their orders of magnitude over a 20 nm distance:

- **Bulk Diffusion (Mitchell’s Model):** The Smoluchowski–Einstein equation estimates a 7 ns travel time at a random point on the sphere. However, actual entry into ATP synthase takes orders of magnitude longer due to the need to target the specifically ATP synthase input channel. In fact, the actual probability of randomly ending up in the right place is only several percentage points (increasing the H^+^ transport time up to hundreds of ns).
- **2D Diffusion:** Takes a minimum time of about 10 ns (to a random point on the circle). Being on the same plane greatly increases the entry probability (up to ~30%). Total transport time is tens of ns.
- **ATP Synthase Rows:** Further raises entry probability up to ~50% by aligning targets.
- **Membrane Curvature:** Guides protons rather than leaving movement to random chance, virtually eliminating the possibility of the proton traveling in the wrong direction and cutting arrival time at least in half compared to the absence of curvature.
- **Lipid Interactions:** Negative lipid head groups cause delays in real systems (at least when electrostatically holding a proton near them). However, if lipids wave in sync with protons instead of slowing them down, transport reaches the speed of sound in lipids (at least 100 m/s). As a result, the propagation time is reduced to ~0.2 ns [37].

So, the cristae structure is self-regulated by protonation and can dynamically form or destruct the pathways for proton conduction. At the same time, if the flow of protons exceeds the capabilities of ATP synthases, then, and only in this case, protons will remain on the respirasomes or go to other regions of the membrane (not only to ATP synthase), which can lead to leaks, ROS synthesis and heat release [39]. This allows mitochondria to maintain their metabolic flexibility (no need for rigid contacts between respirasomes and ATP synthases) and induce ROS-associated signaling under stress conditions. It should be noted that boundary IMM contains its own respiratory chains, which are used only for potential-dependent transporters, but not for ATP synthesis.

## 4. Advantages of Microcompartmentalization in Mitochondria

Based on this structural scheme, the concept of microcompartmentalization should be expanded from the traditional purely steric limitation to a broader concept. Microcompartment denotes the area of space in which the movement of the considered object (a metabolite, protein, or ion) is limited by a potential barrier of any nature (in the case of a proton, for example, an electrodynamic barrier). The thermodynamic and kinetic advantages of individual microcomponents can be formulated as follows:

1. **Localized Proton Motive Force (∆*p*):** Under the classical paradigm, protons pumped into the intracristal space (ICS) would dissipate freely into the bulk IMS, effectively diluting the H^+^ electrochemical gradient. By contrast, sequestering protons within a restricted microcompartment allows the mitochondrion to maintain a high proton flow directly to the ATP synthases. The anisotropic electrical potential in mitochondria is already validated [40], as well as their peri-membrane localization [41]. The functional “local” volume could drop from the attoliter scale to an even more infinitesimal sub-compartment. In such a highly restricted space, even a few protons generate a massive local electrochemical gradient, inducing polarization of the media. Based on this, the IMM should be treated as a supercapacitor, in which the energy is stored and transported at the perimembrane double electric layer or even by the electret principle [42]. This provides both stability and drastically increased electric capacity.
2. **Kinetic Coupling (Substrate Channeling):** When the ICS functions as an isolated compartment, the physical diffusion distance for mobile electron carriers and localized metabolic intermediates is drastically minimized. In some types of mitochondria, for example, in cardiac ones, not only are microcompartments formed as ICS, but the regions of the matrix are trapped by the inner membrane, forming regions where dehydrogenase complexes are sterically clamped. This restriction creates a pronounced “confinement effect.” Rather than diffusing randomly throughout the vast global IMS or matrix, the local concentrations of all metabolites increase dramatically. This spatial confinement ensures that the overall rate of respiration is governed by enzymatic turnover rather than being restricted by the kinetics of three-dimensional diffusion. It also increases the saturation of the active centers of the enzymes, eliminating the problem of high K_m_ in conditions of substrate deficiency.
3. **Buffering Against Osmotic and Ionic Fluctuations:** The outer mitochondrial membrane is relatively porous due to the presence of large voltage-dependent anion channels (VDACs), making the peripheral intermembrane space highly sensitive to fluctuating conditions within the cytoplasm. The cristae junctions act as dynamic “diffusion bottlenecks.” By separating the intracristal space (ICS) from the bulk IMS, the mitochondrion protects the delicate catalytic machinery of the respirasomes from sudden shifts in cytoplasmic pH or ionic strength, maintaining a stable microenvironment that is strictly optimized for oxidative phosphorylation. Cristae junctions can also restrict the diffusion of large molecules such as cytochrome c and other ICS proteins [5].
4. **Redundancy and Safety:** If a single crista—or even an isolated, partitioned segment of a crista—suffers membrane leakage or localized oxidative damage, the collapse of the chemiosmotic potential is entirely contained. The remaining cristae within the mitochondrion continue to function at full capacity. Moreover, recent data shows that compartmentalization of the IMM allows the cell to use microautophagy to locally and selectively remove cristae that have been damaged by oxidative stress [43]. This localized resilience likely explains why mitochondria can withstand high levels of oxidative stress before the entire organelle fails.

## 5. Compartmentalization of NADPH Generation

Unlike NADH, which mainly works in catabolism, NADPH is required for anabolism and antioxidant defense. A critical analysis of cellular bioenergetics reveals that intracellular NADPH pools are not well-mixed. Importantly, the oxidative phosphorylation system is not the only metabolic pathway that becomes more logical and coherent when recognizing functioning in independent microspaces—this view is also valid for other metabolic pathways, known as metabolons. Most cytosolic sources of NADPH are strictly sequestered within their respective metabolic pathways, rendering them incapable of serving as systemic exporters of reducing equivalents. The compartmentalization and sequestration of alternative NADPH sources include the following methods:

- **The Pentose Phosphate Pathway (PPP):** Driven by glucose-6-phosphate dehydrogenase (G-6-PD) and 6-phosphogluconate dehydrogenase (6-PiGDH), the PPP is metabolically tethered to the glycolytic flux and nucleotide biosynthesis [44]. The generated NADPH is immediately consumed, leaving no surplus for systemic distribution.
- **The Malic Enzyme Pathway:** Cytosolic NADP-dependent malic enzyme operates as an obligate component of the citrate–pyruvate shuttle [45]. The reducing equivalents generated by malic enzyme are immediately consumed “on-site” by fatty acid synthase (FAS), preventing any significant net “export” of NADPH.
- **The Cytosolic Folate Cycle:** Its flux is tightly constrained within the one-carbon metabolic pathway [46], rendering it unavailable for general cellular defense.

In that context, the mitochondrial synthesis of NAD(P)H can be discussed.

## 6. Proton-Carrying Nicotinamide Adenine Dinucleotide Transhydrogenase

ATP does not only carry the chemical energy generated by the mitochondria. It can also produce approximately equivalent amounts of NADPH, which serves as the primary carrier of reducing equivalents for anabolic synthesis and antioxidant defense pathways [47,48]. In fact, proton-carrying transhydrogenase plays a role by producing high-energy NADPH, which is, in many ways, similar to ATP synthase [49]. Accordingly, the activity of the enzymes transporting NADPH from mitochondria is about 10 times higher than their reverse activity [50], while the rate of NADPH production in activated submitochondrial particles is only slightly lower than the rate of ATP synthesis [51].

The energy-transducing nicotinamide adenine dinucleotide transhydrogenases of mammalian mitochondria and bacteria are structurally related, membrane-bound enzymes. They catalyze the direct transfer of a hydride ion between NAD(H) and NADP(H) in a reaction coupled with transmembrane proton translocation [47,52,53]. For simplicity, we will refer to this enzyme as “transhydrogenase” (TH). Since this enzyme is much less widely known than the OXPHOS proteins, we will briefly describe its structure and functions below.

TH is an integral membrane enzyme that, under physiological conditions, utilizes the proton-motive force to drive hydride transfer from NADH to NADP^+^. This process is reversible, at least in vitro:

NADH + NADP^+^ + H^+^_out_ ↔ NAD^+^ + NADPH + H^+^_in_.

where H^+^_out_ and H^+^_in_ denote the proton from the intermembrane space and that from the matrix respectively.

In physiological steady-state, TH acts in the forward direction, but under severe energy stress with membrane depolarization, it reverses. In the presence of NADPH, the hydride will be transferred from NADPH to NAD^+^, thereby generating a proton-motive force [47]. Despite the nearly identical standard redox potentials of the NAD^+^/NADH and NADP^+^/NADPH couples, under the physiological conditions in the mitochondrial matrix, electron transfer from NADPH to NAD^+^ is energetically favorable. This is because the actual potential is given by Equation [47]:

(1) $${\Delta E}_{h}=E_{h}\left(NAD\right)-E_{h}\left(NADP\right)=\frac{RT}{2F}ln\left(\frac{a_{{NAD}^{+}}\cdot a_{NADPH}}{a_{NADH}\cdot a_{{NADP}^{+}}}\right)$$

where *a* denotes the activities of the respective species.

Under real conditions of mitochondrial matrix, taking into account a typical NAD^+^/NADH ratio of about 10 and an NADP^+^/NADPH ratio of about 0.01, and using the approximating activities as concentrations, Equation (1) at 37 °C yields ΔE_h_ ≈ 92 mV. That is why, under the depolarization of mitochondria, the TH activity will not allow the potential to fall below this level for some time. This is important because it prevents a complete drop in potential in cases of substrate deficiency, and thus allows the potential-dependent transporters that are necessary to overcome this deficiency to continue working.

**Figure 5:**
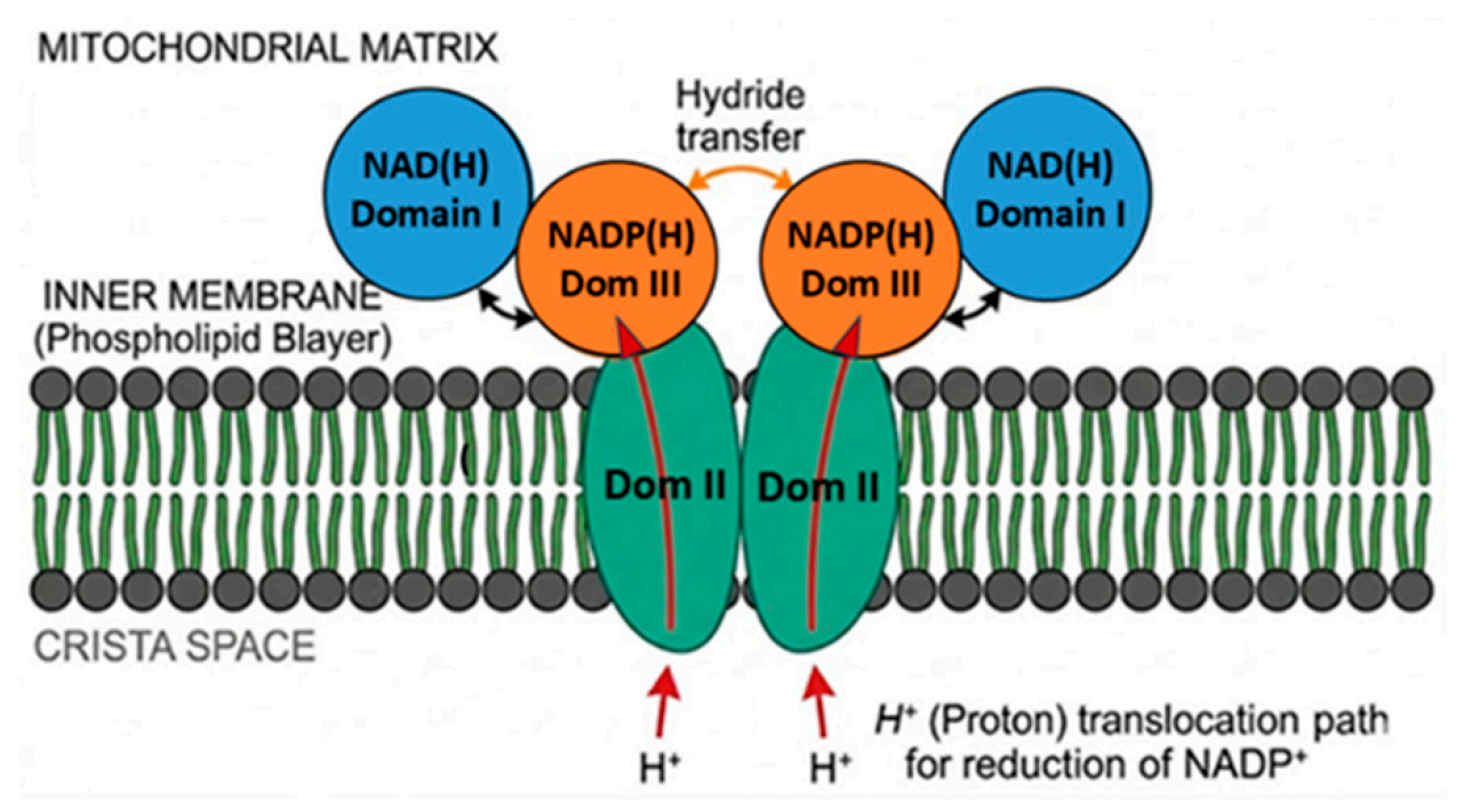
The schematic structure of transhydrogenase.

Structural studies have shown that the active sites of TH, which contain the binding sites for NAD(H) and NADP(H), are located in the same compartment—the mitochondrial matrix [54]. The structural architecture of the transhydrogenase complex is characterized by distinct spatial domains, where the proton-conducting domains (domain II) span the inner membrane as integral membrane proteins. The soluble domains I and III face the mitochondrial matrix and include a 40 kDa-binding domain (domain I) and a 20 kDa-binding domain (domain III), which together catalyze the hydride transfer reaction [55,56]. Crystal structures have revealed two distinct orientations of domain III—designated “face-up” and “face-down”—with respect to the binding sites [55]. The structure of TH is schematically shown in Figure 5.

## 7. TH-ICDH Axis: A Unique “Pipeline” Between the Mitochondrion and the Cytosol

A primary conceptual question arises from the localization of these components: if both the NADH donor and the acceptor NADP^+^ are located within the same compartment (the matrix), what is the physiological significance of this reaction? Furthermore, how is NADPH exported to the cytoplasm? Because mitochondria possess two isoforms of the NADP-dependent isocitrate dehydrogenase (NADP-ICDH) that catalyze reversible oxidation and the reduction in isocitrate, it has been proposed that the physiological function of TH should be evaluated in relation to these ICDH isoforms [47,48].

In humans, ICDH exists in three isoforms. ICDH1 and ICDH2 catalyze reversible reactions and have no known allosteric modifiers. ICDH1 and ICDH2 share considerable sequence similarity (70% identity in humans) and catalyze the same reaction [57]. ICDH3 catalyzes the third irreversible step of the citric acid cycle, converting NAD^+^ to NADH in the mitochondria. ICDH3 is allosterically regulated by a variety of positive (calcium, ADP, and citrate) and negative (ATP, NADH, and NADPH) effectors. The NAD-ICDH-3 heterodimers are composed of αβ- and αγ-subunits, which assemble to form α2βγ heterotetramers [58,59]. ICDH1 is localized in the cytoplasm and peroxisomes, whereas ICDH2 and ICDH3 are found in the matrix of the mitochondria.

The physiological roles of mitochondrial transhydrogenase (TH) and NADP-dependent isocitrate dehydrogenase (NADP-ICDH) have been historically debated. Two primary hypotheses attempt to explain their high activity, particularly in tissues with low NADPH demand (like heart and muscle). First, the hypothesis of Sazanov and Jackson [48] proposes TCA cycle fine-tuning: TH and NADP-ICDH engage in substrate cycling with NAD-ICDH to increase the TCA cycle’s sensitivity to energy demand fluctuations. Second, the hypothesis of Hoek and Rydström [47] suggests energy and nucleotide buffering: TH acts as an “energy buffer” (consuming NADPH to maintain membrane potential during energy depletion) and a “nucleotide buffer” to rapidly adapt to sudden NADPH surges.

Despite these theories, it remains difficult to justify the evolutionary advantage of such a complex control system or explain why both TH and the significantly faster NADP-ICDH [60] are required. Although some details of the precise mechanism of NADPH transport from the matrix to the cytoplasm remained unclear, evidence of the physiological and pathogenetic roles of NADPH begins to emerge [49,61], supporting the need for the strict control and compartmentalization of NADPH.

**Figure 6:**
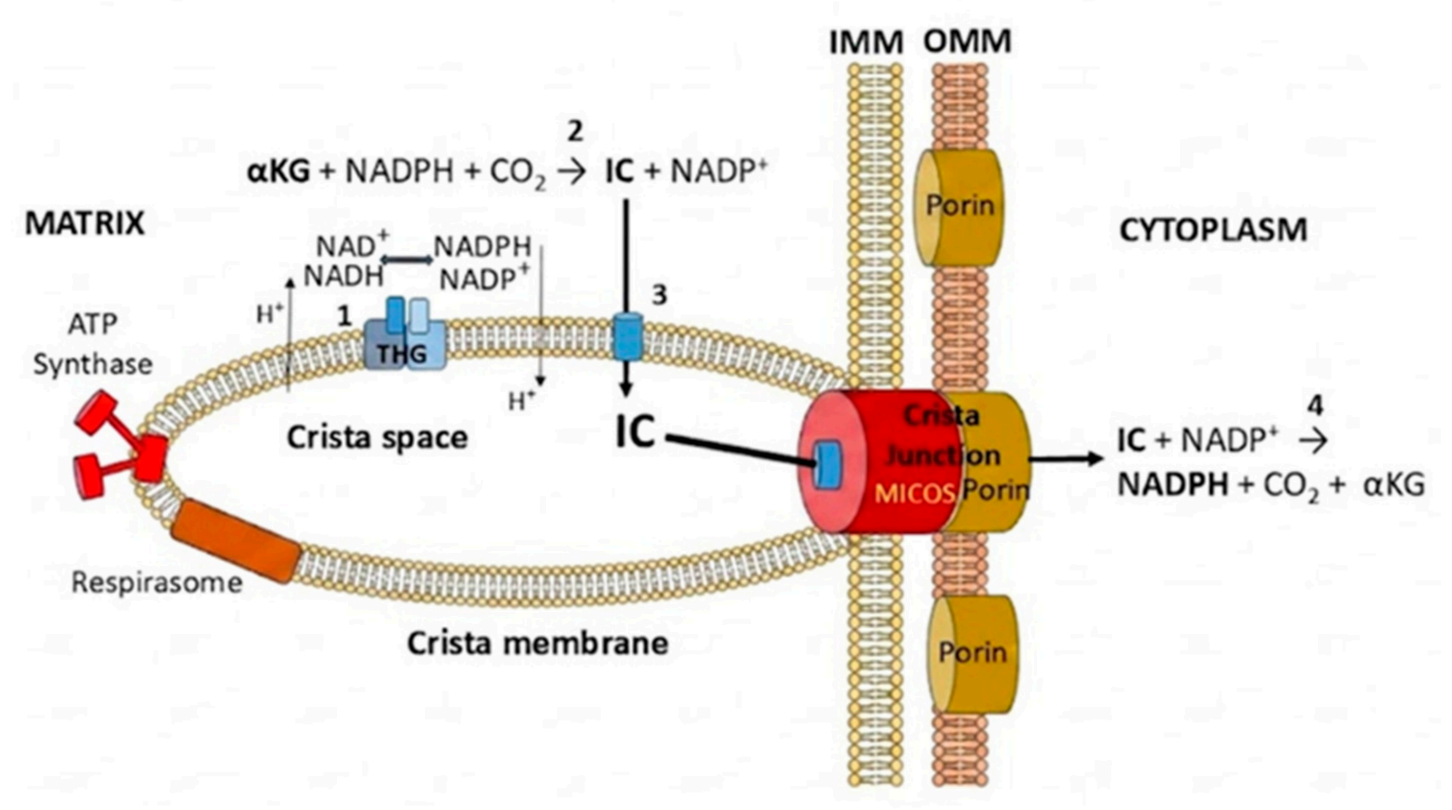
The multistage transfer of NADPH from the mitochondrial matrix to cytoplasm. In the energized mitochondria, all stages are irreversible. Abbreviations: IMM—inner mitochondrial membrane; OMM—outer mitochondrial membrane; αKG—α-ketoglutarate; IC—isocitrate.

According to the latest view, the mitochondria–cytosol NADPH transport (schematically illustrated in Figure 6) contains several stages, involving specific enzymes and transporters:

1. Generation in the Matrix: TH uses a proton gradient to produce NADPH in the matrix.
2. Conversion to Metabolite: Since NADPH cannot freely flow out through the membrane, mitochondrial isocitrate dehydrogenase 2 (ICDH2, matrix isoform) converts it by attaching it to α-ketoglutarate (α-KG):

α-KG + NADPH + CO_2_ → isocitrate + NADP^+^

3. Transport Across the Cristae Membrane: Isocitrate is taken up by the tricarboxylate transporter (TCC, *SLC25A1* gene) embedded in the cristae membrane. This antiporter pumps isocitrate into the microscopic lumen of the crista.
4. Exit into the Cytoplasm and Reverse Conversion: From the cristae, isocitrate exits into the cytoplasm through cristae junctions and porines (VDAC). There, cytosolic isocitrate dehydrogenase 1 (ICDH1) carries out the reverse reaction, generating NADPH in the cytosol:

isocitrate + NADP^+^ → α-KG + NADPH + CO_2_

In more detail, this reaction involves the oxidation of isocitrate to oxalosuccinate, with NAD(P)^+^ as the electron acceptor, followed by the decarboxylation of oxalosuccinate to form a-ketoglutarate [58].

This TH-ICDH loop creates a continuous translocation of reducing equivalents from the mitochondrial matrix to the cytoplasm. The fundamental driving force is thermodynamic pressure, predetermined by a massive increase in entropy as substrates transition from the ultra-confined mitochondrial matrix and intracristal lumen into the exponentially larger volume of the cytoplasm.

## 8. Physiological Significance of NADPH and the NADPH-Generating Enzymes

NADPH serves as the primary currency for cellular reduction, fueling lipid biosynthesis and neutralizing reactive oxygen species (ROS) to protect against oxidative stress. While major metabolic routes channel NADPH directly into specific local metabolic complexes (“committed” NADPH), alternative systems like the transhydrogenase–isocitrate pathway generate a flexible, “uncommitted” pool that diffuses freely to support diverse NADPH-dependent enzymes across the cytoplasm.

**Key cytoplasmic enzymes generating the committed NADPH are as follows** [62]:

- **Glucose-6-Phosphate Dehydrogenase (G6PD):** The rate-limiting enzyme of the oxidative Pentose Phosphate Pathway (PPP), oxidizing glucose-6-phosphate to produce NADPH for peroxide detoxification.
- **6-Phosphogluconate Dehydrogenase (6PGD):** The second NADPH-generating enzyme in the PPP, converting 6-phosphogluconate into ribulose-5-phosphate.
- **NADP^+^-Dependent Malic Enzyme 1 (ME1/Malate Dehydrogenase):** This converts malate to pyruvate in the cytosol, releasing ${\mathrm{CO}}_{2}$ and reducing ${\mathrm{NADP}}^{+}$ to NADPH for use during lipogenesis.
- **Matrix Transhydrogenase–Isocitrate Dehydrogenases Pathway for Generation of the Uncommitted NADPH:**
- **Matrix Nicotinamide Nucleotide Transhydrogenase (NNT):** Driven by the proton-motive force (Δp), NNT transfers hydride ions from NADH to ${\mathrm{NADP}}^{+}$, channeling mitochondrial reducing equivalents into matrix NADPH.
- **Matrix Isocitrate Dehydrogenase 2 (IDH2):** This consumes matrix NADPH via reductive carboxylation to convert α-ketoglutarate and ${\mathrm{CO}}_{2}$ into isocitrate.
- **Cristae Membrane Tricarboxylate Carrier (CIC/*SLC25A1*):** This exports isocitrate across the inner mitochondrial membrane into the cristae space, from where it diffuses into the cytoplasm down the concentration gradient.
- **Cytosolic Isocitrate Dehydrogenase 1 (IDH1):** This oxidizes cytosolic isocitrate back to α-ketoglutarate, generating a pool of cytosolic NADPH that is uncommitted and freely accessible to microsomal oxygenases, biosynthetic enzymes, and antioxidant defense networks. Importantly, this vital source of NADPH is independent of glucose availability.

The metabolic division between pathway-committed and free (uncommitted) NADPH pools represents an elegant natural solution to intracellular transport and compartmentalization challenges. In high-demand pathways like the Pentose Phosphate Pathway (PPP) and fatty acid synthesis, NADPH-generating enzymes are often physically colocalized or embedded in polyenzyme complexes. This spatial arrangement ensures substrate channeling—the generated NADPH is immediately handed off to nearby target enzymes (such as fatty acid synthase or glutathione reductase) without dissipating into the surrounding cytoplasm. This direct coupling accelerates local metabolic flux and prevents wasteful cross-talk.

Conversely, cellular metabolic demands for NADPH fluctuate. When cells face sudden oxidative stress or require NADPH for diverse xenobiotic detoxification pathways, relying solely on pathway-linked enzymes is insufficient. The transhydrogenase-isocitrate pathway fills this gap by utilizing cytosolic IDH1 and matrix transhydrogenase and ICDH2 activity to convert surplus metabolic intermediates into a general-purpose, uncommitted pool of NADPH. This unallocated pool acts as a buffer, ensuring that enzymes throughout the cytoplasm can maintain cellular redox balance and biosynthetic capacity even when main metabolic fluxes shift.

## 9. ATP/ADP Exchange Between the Cristae Space and the Cytoplasm

It is important to note that, when taking into account compartmentalization, the transport of adenine nucleotides from the cristae lumen to cytoplasm is no less complex and regulated than the transport of NADPH. Magnesium ions play an important and often underestimated role in ATP synthesis, transport and utilization. For ATP-consuming enzymes, the physiological substrate is not ATP^4−^ but the lasting complex MgATP^2−^.

The adenine nucleotide translocator (ANT) of IMM carries only “pure,” uncomplexed ATP^4−^ and ADP^3−^ [63]. The binding site of ANT has the same affinity to free adenine nucleotides, but the export of ATP^4−^ from the matrix in exchange for ADP^3−^ is electrogenic and energy-favorable (when there is rather high potential in the IMM). This is the first reason for the irreversibility of such transport. The second reason is associated with the exclusion of ATP from binding to ANT by chelating to Mg^2+^ ions.

In the cytosol, where the steady-state concentration of free Mg^2+^ is maintained at approximately 0.5–1.5 mM (while bounded Mg^2+^ may reach 20 mM or even more [64]), the exported ATP^4−^ instantly chelates magnesium ions to form the divalent anions γ-β-MgATP^2−^ and β-α-MgATP^2−^. Because these chelated complexes do not bind to ANT, they are effectively excluded from back-exchange, instead serving as the biologically active forms of ATP for cytosolic enzymes. This Mg^2+^ binding also increases the energetic value for the ATP hydrolysis: $\Delta G_{\mathrm{cytosol}}\approx -13\;\mathrm{to}-14\;\mathrm{kcal}/\mathrm{mol})\;\mathrm{versus}\;\mathrm{the}\;{\Delta G}_{\mathrm{cristae}/\mathrm{matrix}}\approx -7\;\mathrm{to}\;-9\;\mathrm{kcal}/\mathrm{mol}$ [59]. Conversely, ADP has a significantly lower binding affinity for Mg^2+^, meaning that the concentration of free, uncomplexed ADP^3−^ in the cytoplasm remains about 10 times higher than that of free ATP^4−^ [65]. Some fraction of ATP in the matrix works as a magnesium buffer and an activator of ATP synthases (binds to the alpha subunit). If the ATP concentration drops (which is a signal of depolarization accompanied by energy stress) and magnesium binds to ADP instead of ATP, the so-called MgADP inhibition of ATP synthase occurs, preventing the reverse functioning of ATP synthases.

In addition to the above-mentioned mechanisms, which ensure the direction of transport from the mitochondria to the cytosol, there are other mechanisms, implying the subcompartmentalization of enzymes. During the 1980s, it was demonstrated that, in addition to ANT, various tissue-specific kinases are located precisely at the contact sites between the outer and inner mitochondrial membranes (in BIMS).

Most vertebrate tissues express two isoforms of creatine kinase (CK): a dimeric cytoplasmic form and a mitochondrial form. The mitochondrial isoform predominantly exists as an octamer localized directly at the junctions of the outer and inner membranes. Within this intermembrane space domain, the enzyme forms a structural multi-protein complex alongside VDAC and ANT. This localized isoform mainly utilizes the ATP exported by ANT localized in the inner boundary membrane (IBM) to phosphorylate creatine into phosphocreatine. This phosphocreatine then functions as the specific, mobile transport form of mitochondrial energy. Due to CK, ATP is converted into ADP directly. without leaving the mitochondria, additionally ensuring the irreversibility of energy flow and reducing the diffusion time of adenine nucleotides, returning them immediately to the OXPHOS system. Both CK isoenzymes contribute to establishing the large intracellular pools of phosphocreatine found in the skeletal muscle, myocardium, brain, and renal cortex cells, serving as vital temporal energy buffers under ischemic conditions [66].

Another prominent example of converting intracristal ATP into a cytoplasmic metabolic form (as glucose-6-phosphate) is mediated by hexokinase II (HK-II), which can localize on the OMM. Hexokinase II is the predominant isoform expressed in insulin-dependent tissues, such as the heart, skeletal muscle, and adipose tissue, and its expression is significantly upregulated in many highly glycolytic tumors [67]. It is highly probable that HK-II plays a decisive role in the lactate cycle, acting as a substantial concomitant source of ATP within these cells. HK-II mediates another important mechanism of accelerated ATP-ADP recycling in mitochondria.

## 10. A Brief Overview of the Mechanisms by Which Calcium Ions Influence Compartmentalization

In this review, we barely touched upon the role of calcium ions in mitochondrial regulation, given the vast scope of this topic. However, in the context of transport and compartmentalization, it is worth noting that calcium affects several related key aspects simultaneously:

1. Calcium (and other divalent cations) binding affects the internal curvature of cardiolipin [68], which can influence the curvature of the cristae, fusion/fission and proton transfer processes.
2. Calcium activates some mitochondrial dehydrogenases [69] and, as a result, accelerates metabolism and, consequently, boosts ATP and NADPH production.
3. The calcium transporter MICU1 is a component of the cristae junctions (CJ) [6]; therefore, high Ca^2+^ induces transient opening and remodeling of the cristae junctions. This may both accelerate certain transport processes (including Ca^2+^ itself [70]) and be necessary for apoptosis involving the release of cytochrome c.
4. Calcium acts as an activator of the mitochondrial permeability transition pore and also lipid pores, which, at high concentrations, lead to swelling and significant structural reorganization/disruption of the cristae [71].

Calcium effects are concentration-dependent [72] and are subject to additional regulation, making a detailed examination of them a topic suited for a separate review. It should also be noted that mitochondrial energization is important factor for maintaining low intramitochondrial [Ca^2+^]. There are three primary salts of ${\mathrm{Ca}}^{2+}$ and orthophosphoric acid ($H_{3}{\mathrm{PO}}_{4}$): monocalcium phosphate ($\mathrm{Ca}{\left(H_{2}{\mathrm{PO}}_{4}\right)}_{2}\cdot H_{2}O$), which is moderately soluble in water up to 18 g/L (at 30 °C); dicalcium phosphate (${\mathrm{CaHPO}}_{4}\cdot 2H_{2}O$), which is 57 times less soluble than the mono-salt (0.316 g/L); and tricalcium phosphate (${\mathrm{Ca}}_{3}{\left({\mathrm{PO}}_{4}\right)}_{2}$), which is practically insoluble (0.02 g/L)—making it 900 times less soluble than the mono-salt and 15.8 times less soluble than the di-salt [73]. A fourth calcium phosphate salt is hydroxyapatite, though the exact mechanism of its formation remains poorly understood.

At high mitochondrial energization, the ${\mathrm{Ca}}^{2+}$ taken up by mitochondria is retained in a quasi-insoluble form [74,75]. Upon de-energization, tricalcium phosphate degrades into more soluble forms, and the excess free ${\mathrm{Ca}}^{2+}$ neutralizes the negative charges of cardiolipins and of some amino acid residues. This triggers the $F_{0}F_{1}$ subunit of ATP synthase to convert into the mitochondrial permeability transition pore.

However, it is clear from the above that divalent ions—particularly calcium—are key regulators of mitochondrial structure and function, including their influence on the compartmentalization of cristae.

## 11. Conclusions

The transition from the classical “just maximizing surface area” paradigm to the model of discrete mitochondrial nanocompartmentalization fundamentally rewrites the rules of cellular bioenergetics. By recognizing individual cristae as autonomous, ultra-confined nanoreactors rather than passive invaginations, we resolve long-standing paradoxes in chemiosmotic coupling and metabolite transport. The key takeaways of this revised paradigm are as follows:

1. Electrophysical Basis of Localized Proton-Motive Force (PMF): The intracristal space (ICS) operates as an attoliter-scale microcompartment where proton dissipation is physically restricted. Driven by the unique topology of the membrane (negative curvature at ATP synthase dimers) and the specific biophysical properties of cardiolipin, protons are confined to the perimembrane interface. This creates a massive, localized proton motive force that drives rapid ATP synthesis, decoupled from the bulk intermembrane space.
2. Structural Redundancy and Safety: The segregation of cristae via MICOS-dependent junctions ensures that localized oxidative damage or membrane leakage does not lead to global bioenergetic collapse. This microcompartmentalization provides mitochondria with unprecedented resilience against ischemic and oxidative stress.
3. The TH-ICDH Systemic Pipeline: Viewing the mitochondrion through the lens of microcompartments solves the physiological puzzle of NADPH transport. The Transhydrogenase (TH) and NADP-dependent Isocitrate Dehydrogenase (ICDH) operate not as a mere futile cycle, but as a highly coordinated, irreversible metabolic pipeline. Driven by the thermodynamic gradient between the ultra-confined matrix and the expansive cytosol, this axis continuously exports uncommitted reducing equivalents, fueling systemic antioxidant defense and biosynthesis.
4. Pathophysiological Implications: The structural integrity of cristae microcompartments is the ultimate bottleneck of cellular health. Disruptions in cristae architecture—whether through MICOS/OPA1 disassembly, cardiolipin oxidation, or metabolic network collapse—lead to the dissipation of local PMF and the failure of the TH-ICDH pipeline. Consequently, the loss of microcompartmentalization is not a secondary symptom, but a primary pathogenic driver in neurodegeneration, cardiovascular diseases, and aging.

Ultimately, mitochondrial bioenergetics cannot be fully described by classical equilibrium thermodynamics alone. It requires a non-equilibrium framework that integrates nanoscale structural topology, interfacial electrodynamics, and metabolic compartmentalization to explain the efficiency and flexibility of life at the cellular level.

## Data Availability

No new data were created or analyzed in this study. Data sharing is not applicable to this article.
